# Effect of GnRH administration on egg production, hormonal and biochemical profile and ovarian histomorphology of spent naked neck layers

**DOI:** 10.1016/j.psj.2026.106835

**Published:** 2026-03-21

**Authors:** Shahid Khan, Sarzamin Khan, Shabana Naz, Rifat Ullah Khan, Ala Abudabos, Raed M. Al-Atiyat, Ali R. Al Sulaiman, Sohail Ahmad, Ibrahim A. Alhidary

**Affiliations:** a Department of Poultry Science, Faculty of Animal Husbandry and Veterinary Sciences, The University of Agriculture, Peshawar, Pakistan; b Department of Zoology, Government College University, Faisalabad, Pakistan; c Physiology Lab, College of Veterinary Sciences, Faculty of Animal Husbandry and Veterinary Sciences, The University of Agriculture, Peshawar, Pakistan; d Department of Food and Animal Sciences, College of Agriculture, Tennessee State University, Nashville, TN, 37209, USA; e Molecular Genetics, breeding and Biotechnology, Animal Sci. Dep., Agriculture Faculty, Mutah University, Karak, Jordan; f Environmental Protection Technologies Institute, Sustainability and Environment Sector, King Abdulaziz City for Science and Technology, P.O. Box 6086, Riyadh, 11442, Saudi Arabia; g Islamic University of Afghanistan, Kabul, Afghanistan; h Department of Animal Production, College of Food and Agriculture Sciences, King Saud University, Riyadh, Saudi Arabia

**Keywords:** Follicle stimulating hormone, Gonadotropin releasing hormone, Luteinizing hormone, Serum biochemistry

## Abstract

This study evaluated the effects of GnRH administration on egg production, hormonal and biochemical profile and ovarian histomorphology in spent Naked Neck layers. A total of 150 seventy-two-week-old Naked Neck spent layers were assigned to three treatment groups: a control receiving physiological saline (DL0), GnRH administration every three days (DL1), and GnRH administration every seven days (DL2). Egg production significantly increased (P < 0.001) in GnRH-treated groups from week two onward, with DL2 showing the highest production (52.38% at day 42) compared to DL1 (45.71%) and DL0 (15.23%). Follicle-stimulating hormone (FSH) and luteinizing hormone (LH) levels significantly increased (P < 0.001) in GnRH-treated groups. By day 42, FSH levels reached 2.649 IU/ml in DL2, followed by 1.959 IU/ml in DL1, while the control remained at 1.450 IU/ml. Similarly, LH levels peaked at 0.792 IU/ml in DL2, significantly higher than DL1 (0.627 IU/ml) and DL0 (0.355 IU/ml). Ovarian histomorphology revealed significant follicular development in GnRH-treated groups. Overall, GnRH administration improved reproductive performance in spent layers, with weekly injections (DL2) demonstrating the most pronounced effects on egg production and hormonal profiles.

## Introduction

The poultry industry represents one of the most accessible and efficient sources of high-quality animal protein, primarily through the production of eggs and meat (Khalid et al. 2025). Over the past few decades, poultry production has expanded rapidly due to improvements in breeding, nutrition, and management practices. Commercial poultry strains, including broilers and layers, have been genetically developed as specialized hybrids to maximize meat and egg production under intensive production systems (Hafeez et al. 2023; Akram et al., 2025). As a result, poultry meat and eggs have become major components of human diets worldwide, providing an affordable and valuable source of animal protein (Shami et al. 2024; Yu et al. 2024). Despite these advances, several management and physiological challenges remain, particularly in open-sided housing systems where environmental and managerial factors can influence production efficiency (Islam et al. 2021). Furthermore, local indigenous poultry breeds often exhibit lower reproductive performance, characterized by smaller clutch sizes and early onset of molting, which limits their productivity under traditional management systems.

Reproductive activity in vertebrates is primarily regulated by the neuroendocrine system through the action of gonadotropin-releasing hormone (GnRH), which stimulates the synthesis and secretion of gonadotropins from the anterior pituitary gland (Khan et al. 2013). These gonadotropins, including luteinizing hormone (LH) and follicle-stimulating hormone (FSH), play essential roles in the regulation of gonadal development and reproductive function. In poultry, LH is particularly important for sexual maturation and daily egg production, acting in coordination with progesterone and other sex steroids to regulate ovulation and oviposition (Hanafy and Elnesr, 2021). The gonadotroph cells responsible for the secretion of LH and FSH are distributed throughout both the caudal and cephalic lobes of the anterior pituitary gland (Zhao et al., 2023). In addition, structural and functional changes occur in the pituitary gland during different stages of the reproductive cycle, including periods of active egg production and laying cessation, where cellular proliferation and apoptosis may occur (Chen et al. 2024).

Immunohistochemical (IHC) investigations across various species have demonstrated that most gonadotroph cells contain both LH and FSH. In birds, the secretion of LH is primarily controlled by GnRH released from the hypothalamus (Hanafy and Elnesr, 2021). Similar to other vertebrates, reproductive activities in poultry are regulated by the hypothalamic-pituitary-gonadal (HPG) axis, which coordinates hormonal signaling between the hypothalamus, pituitary gland, and gonads. Proper gonadal function depends on the pulsatile release of gonadotropins from the pituitary gland, which is stimulated by GnRH delivered through the hypothalamic–pituitary portal system. Despite the critical role of GnRH in regulating reproductive physiology, relatively limited research has been conducted on the effects of GnRH treatments in domestic chicken strains (Hanafy and Elnesr, 2021).

Failure of normal ovarian activity may arise from several factors, including anatomical abnormalities, nutritional deficiencies, and inadequate management conditions, all of which can impair gonadotropin secretion and prevent the initiation of folliculogenesis (Zhao et al. 2023). In local poultry breeds, particularly spent hens, reduced reproductive performance may be associated with decreased endocrine stimulation and ovarian inactivity. Therefore, the present study aims to evaluate the efficacy of exogenous GnRH analogs in stimulating reproductive hormone release in spent Naked Neck hens to improve the productivity of indigenous poultry breeds. Specifically, this study investigates the effects of GnRH analog administration at different treatment intervals on production performance, reproductive hormone profiles (LH and FSH), and ovarian histomorphological characteristics in spent Naked Neck layers.

## Materials and methods

### Birds husbandry and experimental protocol

This experiment was conducted at the Department of Poultry Science, Faculty of Animal Husbandry and Veterinary Sciences, The University of Agriculture, Peshawar, Pakistan. Seventy-two-week-old Naked Neck spent layers were selected for the study. Birds were housed in an open-sided poultry shed under semi-controlled environmental conditions. During the trial, the ambient temperature ranged from a minimum of 22°C to a maximum of 32°C, with relative humidity maintained between 55% and 70%. The birds were kept on the floor with a 3 to 4-inch layer of clean, dry litter (bedding material) throughout the experimental period. The study lasted for six weeks. A standard feed was provided at the rate of 110 g per bird per day, while clean drinking water was available ad libitum. A lighting schedule of 16 hours light and 8 hours darkness was maintained daily to support egg production (Table 1) as described by Kyere et al. (2025).

**Table 1:** Formula and chemical composition of the basal Diet (as-fed basis).

Ingredients (%)	Amount
Corn	51.60
Soybean meal (46% CP)	17.40
Wheat grain	10.50
Wheat bran	7.4
Corn gluten meal	2.10
Animal fat	4.50
Limestone	5.10
Dicalcium phosphate (18% P)	1.50
Salt	0.30
DL-Methionine (50%)	0.10
Vitamin premix¹	0.10
Trace mineral premix²	0.10
Calculated Nutrient Composition	
Metabolizable Energy (kcal/kg)	2,950
Crude Protein (%)	16.02
Calcium (%)	3.45
Available Phosphorus (%)	0.81
Methionine + Cystine (%)	0.66
Lysine (%)	0.63

Vitamin premix provided per kg of diet:125,000 IU Vitamin A, 2,500 IU Vitamin D₃, 10 mg Vitamin E, 2 mg Vitamin K₃, 1 mg Vitamin B₁, 5 mg Vitamin B₂, 1 mg Vitamin B₆, 15 mg Vitamin B₁₂, 500 mg Folic Acid, 35,000 mg Niascin, 10,000 mg Ca-Pantothenate, and 50 mg Biotin.

² Trace mineral premix provided per kg of diet:8 mg Manganese (MnO₂), 60 mg Zinc (ZnSO₄), 5 mg Copper (CuSO₄·5H₂O), 40 mg Iron (FeSO₄·7H₂O), 0.3 mg Cobalt (CoSO₄·5H₂O), 1.5 mg Iodine (KI), and 0.15 mg Selenium (Na₂SeO₃·5H₂O).

### GnRH treatment

The GnRH analog used in this study was Dalmarelin® injectable solution, a veterinary pharmaceutical product containing the synthetic gonadotropin-releasing hormone analog Lecirelin acetate as the active ingredient. The product was obtained as a ready-to-use sterile injectable formulation (2 mL vial) with an active ingredient concentration of 0.0262 mg/mL Lecirelin acetate. This GnRH analog stimulates the release of follicle-stimulating hormone (FSH) and luteinizing hormone (LH) from the anterior pituitary gland, thereby activating the hypothalamic–pituitary–gonadal axis and promoting reproductive activity.

### Experimental design

A total of 150 spent Naked Neck hens were randomly and evenly assigned to three treatment groups, with five replicates per group. The treatments (TRT) involved six weeks of intramuscular (i.m.) injections of a GnRH analogue solution, administered either every third day or weekly at a dose of 0.03 mL per injection. Control birds received weekly i.m. injections of 0.15 mL of physiological saline.

### Production performance

Eggs were collected daily from each replicate group, and the total number of eggs was recorded. Daily egg production was calculated based on the hen-day production percentage.

Hen-Day Egg Production (%) = (Total Number of Eggs Produced ÷ Total Number of Live Hens × Number of Days) × 100

### Serum biochemistry

Blood samples were collected weekly from two birds per replicate in each experimental group through the brachial (wing) vein, resulting in six birds sampled per treatment group. Approximately 3 mL of blood per bird was collected using sterile disposable syringes and transferred into plain vacutainer tubes without anticoagulant. The samples were allowed to clot at room temperature for approximately 1 hour, after which they were centrifuged at 3,000 rpm for 15 minutes to separate the serum. The obtained serum was carefully aliquoted into sterile microtubes and stored at −20 °C for up to four weeks until biochemical analysis was performed. Serum biochemical parameters, including total cholesterol, albumin, and total protein, were determined using commercial colorimetric diagnostic kits (BioCheck Inc., UK) following the manufacturer’s instructions. The biochemical analyses were performed in the laboratory by the research team using standard procedures. Absorbance readings were measured using a UV–visible spectrophotometer (Shimadzu UV-1800, Japan). Serum globulin concentration was calculated indirectly by subtracting albumin values from the corresponding total protein concentrations.

### Histomorphology of ovaries

For histological analysis, three birds were randomly selected from each replicate of every experimental group at the end of the experimental period. Birds were selected based on representative body condition and health status, ensuring that they were free from visible disease or physical abnormalities to avoid bias in tissue evaluation. Ovarian tissues were carefully collected immediately after slaughter and fixed for histological processing. The samples were dehydrated through a graded series of ethanol solutions with increasing concentrations (30% to absolute alcohol), cleared in xylene, and embedded in paraffin wax. Thin sections of 3–5 µm thickness were prepared using a rotary microtome. The sections were mounted on glass slides coated with egg albumin to enhance adhesion and were subsequently dried in an oven at 60°C for one hour.

The prepared sections were stained using the Harris hematoxylin and eosin (H&E) staining method. After staining, the slides were cleared in xylene, and a drop of Distrene Plasticizer Xylene (DPX) mounting medium was applied before placing the coverslip.

Finally, the prepared slides were examined under a light microscope at appropriate magnifications by trained personnel in the histology laboratory to evaluate ovarian histomorphological characteristics.

### Statistical analysis

All experimental data were analyzed using the General Linear Model (GLM) procedure of the Statistical Package for the Social Sciences (SPSS version 21.0). The experimental design consisted of three treatment groups (DL0, DL1, and DL2) with repeated measurements over time. Therefore, the effects of treatment, sampling time (days), and their interaction (treatment × time) were evaluated using two-way analysis of variance (ANOVA). Results were expressed as mean ± standard error (SE). When significant differences were detected among treatments, Tukey’s post hoc multiple comparison test was applied to separate the treatment means. Differences were considered statistically significant at P < 0.05. In addition, baseline measurements (day 0) were analyzed separately to confirm the absence of significant differences among experimental groups prior to GnRH administration.

## Results

The results of egg production performance are presented in Table 2. No significant differences were observed among groups on day 7. From day 14 onward, birds treated with GnRH (Dalmiralin) showed significantly higher egg production compared to the control group. The group injected every 7 days consistently recorded the highest egg production, followed by the group injected every 3 days, while the control group showed the lowest performance throughout the experimental period.

**Table 2:** Day egg production performance of laying hens treated with different levels of GnRH hormone.

Days	DL0	DL1	DL2	P value
7	12.38 ±0.88	16.19 ± 0.33	15.04 ± 0.67	0.34
14	15.23^b^± 0.32	28.57^a^± 0.57	34.28^a^± 1.15	0.01
21	16.19^b^± 0.66	37.14^a^± 0.57	42.85^a^± 0.57	0.01
28	14.28^b^± 0.57	41.90^a^± 0.33	46.12^a^± 0.66	0.01
35	13.33^b^± 0.33	44.76^a^± 0.33	49.52^a^± 1.20	0.01
42	15.23^c^± 0.33	45.71^b^± 0.57	52.38^a^± 0.88	0.01

a ^-c^, means with different letters within the row differs at P < 0.05; P < 0.001 LOS, Level of significance, NS, Non-significant DL0 is control, (No injection of Dalmiralin) whereas in the other DL1 (Dalmiralin with interval of 3 days), DL2 (Dalmiralin with interval of 7 days) were injected to the birds intramuscularly. Days (weekly), GnRH (Dalmiralin) level (0.03ml). Egg production (HD %).

The results for serum FSH levels are shown in Table 3. There were no significant differences (P > 0.05) among groups at day 0. However, from day 7 onward, a significant increase (P < 0.001) in serum FSH concentrations was observed in birds treated with GnRH compared to the control group. Birds injected every 7 days showed the highest FSH levels, followed by those injected every 3 days, while the control group consistently recorded the lowest FSH concentrations throughout the study.

**Table 3:** Serum Hormonal profile of FSH of spent layer in response to GnRH administration.

Days	DLO	DL1	DL2	LOS
0	1.451^a^± 0.02	1.516^a^± 0.01	1.445^a^± 0.03	NS
7	1.451^c^± 0.02	1.649^b^± 0.01	2.050^a^± 0.01	0.01
14	1.444^c^± 0.01	1.755^b^± 0.02	2.057^a^± 0.01	0.01
21	1.451^c^± 0.01	1.848^b^± 0.01	2.245^a^± 0.01	0.01
28	1.444^c^± 0.01	1.845^b^± 0.01	2.369^a^±0.07	0.01
35	1.458^c^± 0.02	1.955^b^± 0.01	2.449^a^± 0.01	0.01
42	1.450^c^± 0.01	1.959^b^± 0.01	2.649^a^± 0.01	0.01

a ^-c^, means with different letters within the row differs at P < 0.05; LOS, Level of significance, NS, Non-significant DL0 is control, (No injection of Dalmiralin) whereas in the other DL1 (Dalmiralin with interval of 3 days). DL2 (Dalmiralin with interval of 7 days) were injected to the birds intramuscularly. Days (weekly), GnRH (Dalmiralin) level (0.03ml). FSH (Follicle Stimulating Hormone). FSH values (IU/ml).

The results for serum LH levels are presented in Table 4. No significant differences (P > 0.05) were observed among the groups at day 0. From day 7 onwards, serum LH concentrations significantly increased (P < 0.001) in the GnRH-treated groups compared to the control. The group injected every 7 days consistently showed the highest LH levels, followed by the group injected every 3 days, while the control group maintained the lowest LH concentrations throughout the study period.

**Table 4:** Serum Hormone profile of LHof spent layer in response to GnRH administration.

Days	DLO	DL1	DL2	P value
0	0.351^a^± 0.02	0.358^a^± 0.01	0.353^a^± 0.02	0.32
7	0.356^c^± 0.02	0.47^b^± 0.02	0.667^a^± 0.02	0.02
14	0.352^c^± 0.018	0.515^b^± 0.011	0.692^a^± 0.02	0.01
21	0.355^c^±0.01	0.540^b^±0.010	0.713^a^± 0.01	0.01
28	0.346^c^± 0.01	0.570^b^±0.005	0.736^a^± 0.02	0.01
35	0.351^c^± 0.025	0.592^b^±.0047	0.747^a^± 0.02	0.01
42	0.355^c^± 0.017	0.627^b^± 0.0332	0.792^a^±0.002	0.01

a ^-c^means with different letters within the row differs at P < 0.05; DL0 is control, (No injection of Dalmiralin) whereas in the other DL1 (Dalmiralin with interval of 3 days). DL2 (Dalmiralin with interval of 7 days) were injected to the birds intramuscularly. Days (weekly), GnRH (Dalmiralin) level (0.03ml). LH (Luteinizing Hormone). LH values (IU/ml).

The results for serum cholesterol levels are presented in Table 5. No significant differences (P > 0.05) were observed among groups throughout most of the experimental period. However, on day 42, a numerical increase in cholesterol levels was observed in GnRH-treated birds, with the group injected every 7 days recording the highest values, but this difference was not statistically significant (P > 0.05). Overall, GnRH administration had no significant effect on serum cholesterol levels during the study.

**Table 5:** Serum Cholesterolof spent layer in response to GnRH administration.

Days	DLO	DL1	DL2	P value
7	110.33^b^± 1.18	112.01^a^^b^± 1.06	114.03 ± 0.92	0.123
14	110.41^a^± 1.45	111.77^a^± 2.55	109.10^a^± 1.68	0.649
21	108.52^a^± 2.17	106.82^a^± 1.38	106.03^a^± 2.19	0.674
28	105.87^a^± 1.21	106.93^a^± 3.15	110.23^a^± 1.53	0.383
35	105.27^a^± 0.54	108.75^a^± 2.20	107.82^a^± 2.34	0.448
42	107.09^b^± 1.63	109.93^a^^b^± 1.42	112.20^a^± 1.30	0.120

a ^bc^, means with different letters within the row did not differs at P < 0.05; DL0 is control, (No injection of Dalmiralin) whereas in the other DL1 (Dalmiralin with interval of 3 days). DL2 (Dalmiralin with interval of 7 days) were injected to the birds intramuscularly. Days (weekly), GnRH (Dalmiralin) level (0.03ml). #Serum cholesterol (mg/dl).

The results for serum total protein levels are presented in Table 6. No significant differences (P > 0.05) were observed among groups from day 7 to day 35. However, a numerical increase in total protein levels was recorded in the GnRH-treated birds, particularly in the group injected every 7 days. On day 42, birds receiving GnRH showed a significant increase (P = 0.021) in serum total protein, with the highest levels observed in birds injected at 7-day intervals, followed by those injected every 3 days, while the control group had the lowest values.

**Table 6:** Serum total protein of spent layer in response to GnRH administration.

Days	DLO	DL1	DL2	P value
7	4.633^b^± 0.31	4.866^a^^b^± 0.28	5.533^a^± 0.12^a^	0.106
14	4.766^a^± 0.44	5.233^a^± 0.29	5.466^a^± 0.1^a^	0.346
21	5.066^a^± 0.40	4.900^a^± 0.15	4.533^a^± 0.14^a^	0.405
28	4.800^a^± 0.17	5.233^a^± 0.24	5.366^a^± 0.14^a^	0.169
35	5.466^b^± 0.18	5.900^a^^b^± 0.11	6.000^a^± 0.15^a^	0.103
42	5.433^b^± 0.08	6.100^a^± 0.11	6.266^a^± 0.23^a^	0.021

a ^bc^, means with different letters within the row did not differs at P < 0.05; DL0 is control, (No injection of Dalmiralin) whereas in the other DL1 (Dalmiralin with interval of 3 days). DL2 (Dalmiralin with interval of 7 days) were injected to the birds intramuscularly. Days (weekly), GnRH (Dalmiralin) level (0.03ml). #Serum total protein (g/dl).

The results showed no significant differences (P > 0.05) in serum albumin levels among groups during the experimental period; however, a numerical increase in albumin concentration was observed in the treated groups (DL1 and DL2) compared to the control, with a tendency towards significance on day 42 (P = 0.068) (Table 7).

**Table 7:** Serum albumin of spent layer in response to GnRH administration.

Days	DLO	DL1	DL2	P value
7	2.333 ± 0.06	2.533 ± 0.18	2.600 ± 0.11	0.389
14	2.566 ± 0.29	3.033 ± 0.29	3.232 ± 0.08	0.225
21	2.900 ± 0.36	2.733 ± 0.20	2.434 ± 0.14	0.464
28	2.766 ± 0.14	2.866 ± 0.21	2.833 ± 0.13	0.915
35	3.233 ± 0.32	3.466 ± 0.12	3.365 ± 0.12	0.750
42	3.233^ab^± 0.08	3.533^ab^± 0.08	3.562^a^± 0.08	0.068

a ^bc^, means with different letters within the row did not differs at P < 0.05; DL0 is control, (No injection of Dalmiralin) whereas in the other DL1 (Dalmiralin with interval of 3 days). DL2 (Dalmiralin with interval of 7 days) were injected to the birds intramuscularly. Days (weekly), GnRH (Dalmiralin) level (0.03ml). #Serum albumin (g/dl).

The results revealed no significant differences (P > 0.05) in serum globulin levels among groups throughout the study; however, birds treated with GnRH, particularly in DL2, showed a numerical increase in globulin concentrations with a tendency towards improvement on day 42 (P = 0.108) (Table 8).

**Table 8:** Serum globulin of spent layer in response to GnRH administration.

Days	DLO	DL1	DL2	P value
7	2.300^a^± 0.26	2.333^a^± 0.24	2.933^a^± 0.03	0.127
14	2.200^a^± 0.15	2.200^a^± 0.10	2.233^a^± 0.18	0.983
21	2.166^a^± 0.06	2.166^a^± 0.12	2.100^a^± 0.00	0.797
28	2.033^a^± 0.14	2.366^a^± 0.08	2.533^a^± 0.27	0.232
35	2.233^a^± 0.17	2.433^a^± 0.23	2.633^a^± 0.06	0.331
42	2.200^b^± 0.17	2.566^a^^b^± 0.08	2.700 a± 0.15	0.108

a ^bc^, means with different letters within the row did not differs at P < 0.05; DL0 is control, (No injection of Dalmiralin) whereas in the other DL1 (Dalmiralin with interval of 3 days). DL2 (Dalmiralin with interval of 7 days) were injected to the birds intramuscularly. Days (weekly), GnRH (Dalmiralin) level (0.03ml). #Serum globulin (g/dl).

The histological examination of ovarian tissues from the control and treated groups revealed distinct differences in follicular development and ovarian activity following GnRH administration.

**Fig. 1:**
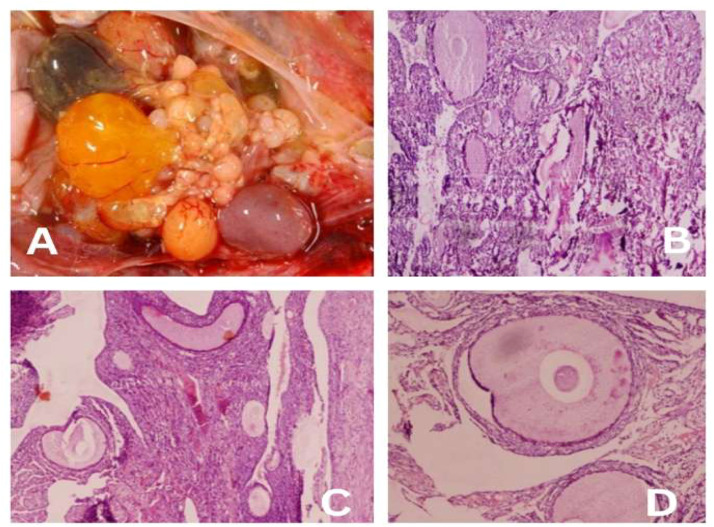
Presence of mature follicles and developed yolk within the shell gland of the DL2 treated group (A), demonstrating that hormonal stimulation activated previously inactive follicles, promoting maturation; Control (B), DL1 (C), DL2 (D).

The treated group demonstrated the presence of mature follicles and egg yolk in the shell gland, suggesting that previously inactive ovarian tissues became active under the influence of exogenously administered GnRH (Fig. 1A). The ovarian tissue of the control group (DL0) exhibited immature follicles or oocytes, indicating that follicular maturation requires time to fully develop in the absence of hormonal stimulation (Fig. 1B).

The histomicrograph of the ovary from the DL1 group showed mature follicles within the cortical region, representing the activation of normal ovarian function in response to hormonal treatment as shown in Fig. 1C. The ovarian histomicrograph of the DL2 group exhibited fully mature follicles or oocytes within the ovarian cortex, indicating a fully active ovary stimulated by exogenous hormone administration as given in Fig. 1D.

## Discussion

The findings revealed that GnRH treatment significantly improved egg production performance, hormonal profiles (FSH and LH), and ovarian histological development, while showing marginal effects on serum biochemical indices such as cholesterol, total protein, albumin, and globulin. The results of egg production indicated that from day 14 onwards, birds treated with GnRH exhibited significantly higher hen-day egg production compared to the control group, with the DL2 group (injected every 7 days) recording the highest values. This aligns with previous reports suggesting that GnRH is a key neuropeptide responsible for regulating reproductive cyclicity by stimulating the pituitary gland to release gonadotropins such as FSH and LH, which in turn promote follicular development, ovulation, and egg production (Hanafy and Elnesr, 2021). The enhanced egg production in GnRH-treated birds may be attributed to the significant elevation of serum FSH and LH levels observed from day 7 onwards (Yang et al. 2024). FSH plays a pivotal role in the recruitment and growth of ovarian follicles, while LH is essential for final follicle maturation and ovulation (). The present study showed a dose-dependent response, with birds injected at 7-day intervals demonstrating superior FSH and LH concentrations compared to those injected every 3 days. This may reflect the pulsatile nature of GnRH action, where appropriate dosing intervals ensure sustained stimulation of gonadotropin release, as supported by studies in poultry and mammals (Zhao et al. 2023).

Histological analysis of ovarian tissues further substantiated these findings, where GnRH-treated groups, particularly DL2, exhibited fully mature follicles and visible yolk deposition in the shell gland (Fig. 1). This clearly indicates that exogenous GnRH effectively reactivated the ovarian axis, transitioning inactive or regressed follicles into functional structures capable of supporting egg production. Similar ovarian reactivation has been reported in spent layers and aging hens following hormonal interventions (Hanafy and Elnesr 2021).

Interestingly, while significant improvements were observed in reproductive parameters, the impact of GnRH administration on serum cholesterol levels remained statistically non-significant throughout most of the experimental period. However, a numerical increase was noted in treated groups, particularly DL2, at day 42. Cholesterol is a precursor for steroid hormone biosynthesis, including estrogens and progesterone, which are essential for follicular growth and ovulation (Wang et al. 2022). Although the observed cholesterol elevation was modest, it reflects the physiological demand for steroidogenesis initiated by GnRH-induced gonadotropin surge.

Total serum protein levels exhibited a numerical increase in GnRH-treated birds, becoming statistically significant at day 42, with the highest values in DL2. Total protein levels, reflecting circulating albumin and globulins, can be influenced by enhanced metabolic activity, improved hepatic function, or increased protein synthesis associated with reproductive activity (Saqib et al. 2022). The delayed but significant rise in total protein levels corresponds with the sustained follicular development and egg production improvements observed during the latter phase of the experiment.

In contrast, serum albumin and globulin concentrations showed numerical but statistically non-significant differences, with tendencies towards improvement on day 42. Albumin is a major plasma protein synthesized by the liver, reflecting nutritional and metabolic status, while globulins are associated with immune function and transport of hormones (Saqib et al., 2018). The upward trend observed in the present study may indicate improved physiological status following GnRH stimulation, although the effects on these parameters were relatively mild compared to reproductive outcomes.

The observed reproductive enhancements are consistent with the mechanisms of GnRH action reported in avian species. GnRH binds to specific receptors in the anterior pituitary, triggering the synthesis and release of FSH and LH, which in turn regulate ovarian folliculogenesis and ovulation (Pawson and McNeilly 2005). Previous studies demonstrated that GnRH analogues or pulsatile administration significantly enhances ovarian activity and egg production in poultry, especially in birds with declining reproductive performance (Hanafy and Elnesr, 2021). Moreover, the superior response observed in the group injected every 7 days aligns with the concept that optimal GnRH dosing intervals prevent receptor desensitization and maintain the physiological rhythm necessary for effective gonadotropin release (Hanafy and Elnesr, 2021). Over-frequent administration, as seen in the 3-day group (DL1), may result in partial receptor downregulation or altered pituitary responsiveness, leading to comparatively lower, though still improved, reproductive parameters relative to the control.

Histological evaluations further corroborated the physiological and biochemical findings. The control group exhibited predominantly immature follicles, suggesting limited ovarian activity, a common feature in spent layers (Khan et al. 2011). In contrast, DL1 and DL2 groups displayed progressive follicular maturation, with DL2 showing complete development of mature oocytes, yolk deposition, and active ovarian architecture (Fig. 1). These morphological changes are directly associated with the hormonal milieu induced by GnRH treatment, demonstrating reactivation of the hypothalamic-pituitary-gonadal (HPG) axis (Hanafy and Elnesr, 2021).

From a practical perspective, these findings highlight the potential of strategic GnRH administration in improving the reproductive efficiency of spent layers, extending their productive lifespan and economic value. The data suggest that a 7-day dosing interval is more effective than a 3-day regimen, providing a foundation for optimizing hormonal intervention protocols in poultry production.

## Conclusion

GnRH administration significantly enhanced egg production, as well as FSH and LH secretion, in spent Naked Neck layers, with the 7-day interval (DL2) showing the most pronounced effect. While serum cholesterol, total protein, albumin, and globulin levels exhibited minor numerical variations, they were not significantly affected by the treatment, suggesting that GnRH primarily influences reproductive performance rather than metabolic parameters.

## Funding

Not available.

## Ethical statement

The study was approved by the Ethical Committee on Animal Rights and Welfare, The University of Agriculture, Peshawar, Pakistan.

## Data availability

The relevant data are provided in the paper. The data of the current experiment can be obtained from corresponding author when needed.

## AI

We used AI (chatgpt) for English correction.

## CRediT authorship contribution statement

**Shahid Khan:** Methodology, Investigation. **Sarzamin Khan:** Supervision, Conceptualization. **Shabana Naz:** Visualization, Validation. **Rifat Ullah Khan:** Investigation, Formal analysis, Data curation. **Ala Abudabos:** Writing – review & editing, Writing – original draft. **Raed M. Al-Atiyat:** Writing – original draft, Writing – review & editing. **Ali R. Al Sulaiman:** Writing – original draft, Writing – review & editing. **Sohail Ahmad:** Writing – review & editing, Writing – original draft, Funding acquisition. **Ibrahim A. Alhidary:** Funding acquisition.

## Disclosures

Authors declare no conflict of interest.

No potential conflict of interest was reported by the author(s).
